# Exploring the involvement of representatives of migrant groups in health research: Elin Lampa

**DOI:** 10.1093/eurpub/ckac129.725

**Published:** 2022-10-25

**Authors:** E Lampa, A Sarkadi, F Osman, A Tökés, N Johansson, G Warner

**Affiliations:** 1 Child Health and Parenting, Uppsala University, Uppsala, Sweden; 2 School of Health and Welfare, Dalarna University, Borlänge, Sweden

## Abstract

**Background:**

Public involvement in research is increasingly utilised, but has been problematised for lack of diversity. Involving representatives from seldom-heard groups, such as the migrant population, has the potential to transform health research for some of Europe's most disadvantaged groups.

**Methods:**

We have explored involvement of migrants in health research projects in Sweden, through a three-year longitudinal qualitative study with migrant contributors involved in a child mental health trial, and a series of behavioural observations of research meetings in several projects with migrant contributors.

**Results:**

The migrant contributors were initially hesitant to trust the researchers. However, through relationship-building and time, mutual trust was established. The contributors gained a social network in each other and the researchers, and their motivation for involvement changed over time, from focussing on individual benefits to societal change. They viewed their role as sharing their experiences, but saw researchers as in control of the final research decisions. Behavioural observations identified barriers to contributors’ access to information in the meetings, such as academic terminology and difficulties in language interpretation. Enabling factors included balancing the presence of experts in the meeting as well as flexibility towards contributors’ needs and initiatives. Additionally, transparency around the research process and providing feedback to the contributors functioned as enablers for involvement.

**Conclusions:**

This longitudinal qualitative inquiry paired with behavioural observations, revealed that when involving migrants as public contributors in research, time and resources should be focused on relationship building, to increase mutual trust and understanding, and careful planning undertaken to make the research process transparent and accessible for the contributors.

**Key messages:**

• Involving migrants in research has great potential – but requires careful planning and consideration.

• Awareness of barriers and enablers can assist researchers in attaining meaningful involvement.

